# Cosmetic usage: Knowledge, attitude and behaviour among female college students in India

**DOI:** 10.6026/973206300211937

**Published:** 2025-07-31

**Authors:** Haritha Senthilvel, Stephen T., Angeline Grace

**Affiliations:** 1 Department of Community Medicine, Sree Balaji Medical College & Hospital, BIHER, Chennai, Tamil Nadu, India

**Keywords:** Cosmetic use, college students, knowledge-attitude-behaviour, public health, adverse effects, beauty practices, cosmetic literacy

## Abstract

Cosmetic use is increasingly common among female college students, influenced by sociocultural and peer factors. Therefore, it is of interest to assessed knowledge, attitudes, behaviours and health implications related to cosmetic use among 382 female arts college students at Chrompet, Chennai, India. Most participants began using cosmetics after age 15, primarily for skincare, with social media being the main influence. Common adverse effects reported were hair damage, skin itching, and colour change. The need for educational interventions and regulatory measures to promote safe cosmetic practices is needed.

## Background:

Cosmetics play a significant role in everyday life for people of all ages, serving various purposes [1]. College students' use of cosmetics has become a common phenomenon worldwide, influenced by a variety of social, cultural and psychological factors. College students, especially female students, frequently use cosmetics to improve their physical appearance, increase their self-confidence and conform to social norms, but this behaviour has consequences because it reflects underlying attitudes, knowledge and behavioural patterns regarding cosmetic products. Research has shown that social prestige, peer pressure and educational attainment all have a significant impact on students' cosmetic usage habits [2]. This aims to explore the multifaceted dimensions of cosmetic usage among college students while highlighting the importance of assessing their knowledge, attitudes and behaviors toward these products. Knowing the components, advantages and possible hazards of cosmetics is part of the knowledge component of using them. Saudi Arabian research found that although 93.4% of female college students reported using cosmetics, there was a notable lack of knowledge about the negative impacts of the chemicals used in cosmetics [3]. Similarly, studies in Ethiopia reported that more than half of female students experienced adverse reactions such as skin rashes and itching due to improper or uninformed use of cosmetics [4]. These findings underscore the need for educational interventions to improve students' knowledge about safe cosmetic practices and product selection. Attitudes toward cosmetics are often shaped by cultural norms and individual perceptions of beauty. In Iran, attitudes and prototypes [social imagination] were identified as strong predictors of cosmetic usage among female students [2]. It was seen that the social status connected to particular academic disciplines, like medicine or pharmacy, had a major impact on cosmetic use. Because of their increased self-confidence and the expectations of society, students in these disciplines typically used cosmetics more regularly [3]. This highlights how attitudes toward cosmetics are intertwined with social identity and cultural values. Depending on demographic and socioeconomic characteristics, there are considerable variations in the behavioural patterns surrounding the use of cosmetics. For example, it was discovered that Ethiopian college students use a range of cosmetics on a regular basis, such as lipstick, toothpaste, deodorant and perfume [4]. However, there was a greater prevalence of cosmetic-related health problems because habits like reading product labels and checking for negative responses were less widespread. Saudi students, on the other hand, used cosmetics frequently every day but lacked the understanding of how to use it safely [3]. These behavioral trends reflect the need for targeted interventions to promote rational cosmetic utilization practices. Assessing people's knowledge, attitudes and behaviours about the use of cosmetics is essential to comprehending its wider effects on society and health.

Hazardous materials including heavy metals and preservatives are frequently found in cosmetic products, endangering the health of users [3]. Frequent cosmetic use among college-aged women is driven by social media and peer influences, which foster body dissatisfaction, appearance anxiety, and lowered self-esteem [14]. The idealised and digitally-enhanced images promoted online-such as filtered selfies-reinforce unrealistic beauty standards and can contribute to self-objectification and body dysmorphic tendencies. Studies indicate substantial knowledge-practice gaps in cosmetic safety among undergraduates: while awareness may be moderate, 35-75 % report mild adverse effects like skin itching, acne, or rashes [15]. Moreover, evidence shows that although makeup use can temporarily boost confidence, it often perpetuates reliance on appearance for self-worth, reinforcing societal beauty pressures. Therefore, it is of interest to describe the knowledge, attitudes and behaviours of female Arts College in India students regarding cosmetic usage and associated health implications.

## Methodology:

## Study design:

Cross-sectional study.

## Study area:

Arts College in Chrompet, Chennai; There are 3 arts college in Chrompet area. One of the colleges among them was selected through simple random sampling.

## Study population:

Female college students

## Inclusion criteria:

Female students aged above 18 and willing to participate in the study.

## Exclusion criteria:

Students with known skin allergies under treatment

## Study period:

November 23 to January 24

## Sample size:

Z = (4 Q/L2 ) [P = 50%, Q = 100 - 6.98,L(Absolute Precision) = 5% ]. The sample size was calculated as 380. P = prevalence of knowledge among college students, d = the degree of precision was at 0.05 at 95% confidence interval and anticipated prevalence of knowledge among college students = 50%

## Sampling method:

A list of female students from the selected Arts College was prepared. 380 students were selected from the list for the study with use of simple random sampling method. The knowledge, attitude and behaviour of selected students were evaluated using the questionnaire.

## Data analysis:

Data was entered in the google forms and analysed using SPSS version 24

## Ethical issue:

Ethical approval was obtained from the institutional review board (IRB) to ensure compliance with ethical guidelines and safeguard participants' rights. Informed consent was obtained from all participants, emphasizing the voluntary nature of their participation and the right to withdraw at any stage without repercussions.

## Results:

The study was conducted among 382 female arts college students. As seen in Table 1 (see PDF), 65.45% of study participants had started their exposure to cosmetics after 15 years. Among the type of care, 73.82% of study participants used skin care as seen in Figure 1 (see PDF). 44.76% of study participants were using cosmetics for special occasions, whereas 22.75% were using to enhance beauty. 31.41% of study participants stated that there are negative effects of usage of cosmetics. On asked about the motivating factor for the usage of cosmetics, 69.90% told they prefer usage by themselves. When enquired about the need for guidelines in usage in educational instidtutions, 36.91% replied yes. As seen in Figure 2 (see PDF), 84% (n= 321) felt comfortable discussing their usage of cosmetics with family & friends. 36.65% of study participants maintained their cosmetics monthly once, whereas 27.75% maintained it once a week. As seen in Table 2, 44.76% (n=171) students told, they were using cosmetics on special occasions, whereas 22.25% [n=85] used it to enhance beauty, when asked about the primary reason for usage of cosmetics. 84% of students said that they were comfortable with discussion about cosmetics as seen in Figure 2 (see PDF). 22.27% of students experienced a change in skin colour after using cosmetics. 49.48% of study participants experienced breakage or damage to hair. 31.15% of students experienced itching after using cosmetics.

## Discussion:

Our study found that female college students begin using cosmetics at a relatively young age, which aligns with global trends of increasing cosmetic use among adolescents and young adults. This early initiation into cosmetic usage merits attention from public health perspectives, particularly regarding product safety awareness. Recent research conducted in the United Arab Emirates by Bashareh *et al.* revealed that college-aged women have significant interest in cosmetic enhancements, with aesthetic appearance being the primary driver [5]. Our study documented various adverse effects experienced by participants, which correlate with findings from other research. 32.9% of study participants experienced side effects with the usage of cosmetics in a study done by Mehta *et al.* [6]; whereas it was 26.4% and 50.6% in studies done by Khan *et al.* [7] and Lucca *et al.* respectively [1]. Udhayanga *et al.* colleagues found that the majority of Sri Lankan students experienced cosmetic-related symptoms such as skin dryness (24.0%), acne (21%), allergies (20.5%) and rashes (19.8%). These data emphasise the incidence of adverse reactions, especially among young, otherwise healthy individuals. Social media contributed to maximum awareness (63.87%) about cosmetic products in our study followed by advertisements (20.42%); whereas in a study done in Turkey by Khan *et al.* friends and families contributed to 31.3% [7]. In another study done among college students in Kashmir by Bhat *et al.* found that electronic media contributed to awareness among 35.5% followed by friends, 33.5% [8].

Research with university students in other locations has demonstrated that a lack of knowledge about cosmetics and their components, often exacerbated by misleading marketing, can lead to misguided judgments with long-term health effects. Despite knowing the risks, many young adults continue to use cosmetic goods since look; image and self-confidence are highly related with these products, according to a study done by Udhayanga *et al.* [9]. Understanding the motivations behind cosmetic usage, whether driven by self-enhancement, social pressure, or other factors, is crucial for designing targeted educational interventions as said by Korichi *et al.* [10]. The understanding of the need for awareness programs demonstrates students' eagerness to learn more about safe and ethical cosmetic methods, creating an opportunity for universities and public health organisations to conduct effective educational activities [11]. To equip customers with the knowledge they need to choose the right products, identify negative responses and adopt safe practices, educational initiatives are crucial. Initiatives such as online classes, for example, have demonstrated efficacy in enhancing skin care routines and lowering health problems associated with cosmetic procedures; participants report improved comprehension and better behaviours [12-13]. These initiatives are an essential part of public health policies since they not only reduce hazards but also encourage educated decision-making. While our research gives useful insights, certain limitations must be addressed. The cross-sectional design provides a snapshot of present behaviours but does not prove causality or track changes over time. Future longitudinal studies could provide further information about how cosmetic usage patterns change during young adulthood and the long-term health consequences.

## Conclusion:

Early cosmetic use and limited awareness of product safety among female college students pose significant public health concerns. Addressing these issues through targeted educational initiatives can promote informed, safer choices. Thus, collaborative efforts between educators, health professionals and regulators are essential to enhance cosmetic literacy and reduce health risks.
